# Hepatic response of male *Astyanax lacustris* (Characiformes: Characidae) to long-term exposure to elevated water temperatures

**DOI:** 10.1007/s10695-026-01827-5

**Published:** 2026-11-05

**Authors:** Bruna Caroline Kotz Kliemann, Vitória Fernanda Martins Do Nascimento, Bruna Mayra Bispo da Silva Braziliano, Cristieli Fernanda Belancieri Souza, Alexandre Ninhaus-Silveira, Rosicleire Veríssimo-Silveira

**Affiliations:** 1 Department of Biology and Animal Science, School of Engineering, Ilha Solteira, Postgraduate Program in Animal Science and Technology, São Paulo State University (UNESP), Monção Street, 226, Zona Norte, São Paulo, Ilha Solteira 15385-086 Brazil; 2 Department of Biology and Animal Science, School of Engineering, Ilha Solteira, Degree in Biological Sciences, São Paulo State University (UNESP), Monção Street, 226, Zona Norte, São Paulo, Ilha Solteira 22615385-086 Brazil

**Keywords:** Water temperature, Freshwater, Liver, Histopathology, Water recycling system

## Abstract

The average global temperature has increased significantly in recent decades due to anthropogenic activities. In aquatic environments, rising temperatures act as a major thermal stressor, affecting fish growth and development. However, little is known about the effects of elevated temperatures on the liver tissue of *Astyanax lacustris*, a species of importance in aquaculture. Thus, this study investigated the effects of high temperature (32 °C) and exposure duration on liver histopathology in *A. lacustris*. Forty males were maintained in a recirculating aquaculture system under two temperatures (26 °C and 32 °C) and evaluated after 30 and 210 days of exposure. Histopathological alterations such as leukocyte infiltration, cytoplasmic vacuolization, and hyperemia were observed at low frequencies, associated with both exposure time and elevated temperature. Vascular congestion, a more severe condition that can compromise liver function, was not directly related to temperature increase; however, it was associated with the reproductive period. The histopathological index indicated mild to moderate damage at 26 °C after 30 days, whereas normal hepatic function was preserved in all other groups. Overall, the low incidence and severity of lesions indicate that *A. lacustris* can maintain liver integrity when exposed to temperatures up to 32 °C in recirculating water systems. These findings suggest a degree of hepatic resilience to warming conditions, although longer-term exposure may increase the risk of tissue alterations, highlighting the need for further investigations under climate change scenarios.

## Introduction

The global average temperature has increased significantly in recent decades due to anthropogenic activities (Stocker 2014; Jeppesen et al. 2015). According to the Intergovernmental Panel on Climate Change (IPCC 2023), global temperatures are projected to increase by approximately 1.5 °C by 2040 and by 3.3–5.7 °C by 2100, depending on future greenhouse gas emission scenarios. In aquatic environments, rising temperatures act as a thermal stressor for organisms, affecting fish growth and development (Albouy et al. 2012; Cox et al. 2018; Islam et al. 2022a, 2022b). Aquaculture productivity and viability may also be impacted by temperature-driven changes, particularly through effects on water quality (Beitinger et al. 2000; Kutty 2005) and fish physiology (Alfonso et al. 2020; Islam et al. 2020), ultimately influencing the performance of fish farms (Uppanunchai et al. 2018; Islam et al. 2020, 2022a, 2022b). Therefore, understanding fish’s physiological responses to temperature variation is essential for the aquaculture sector. Despite its importance, little is known about the effects of elevated temperatures on the liver tissue of *Astyanax lacustris* (Lütken, 1875), a species of relevance in aquaculture (Sabbag et al. 2011).

*Astyanax lacustris* (previously described as *Astyanax altiparanae*; Lucena and Soares 2016) has been widely used in aquaculture and as an experimental model due to its adaptability and suitability for captive conditions (Porto-Foresti et al. 2001; Orsi et al. 2004; Vidotto-Magnoni et al. 2021). This species is a member of the family Characidae, with a small body, a great ability to adapt to different habitats, rapid reproduction, and is considered a trophic generalist. Characidae tend to be more susceptible to increases in environmental temperature due to their higher physiological activity, as indicated by higher caudal fin aspect ratios and metabolic rates (Campos et al. 2018). According to Pereira et al. (2022), tolerance and acclimation to new thermal conditions are more challenging for active species than for sedentary ones. In such species, the higher energy expenditure required to sustain an active metabolism can limit the energy available to cope with thermal variation (Campos et al. 2019).

As ectothermic organisms that are unable to produce their own heat, fish can tolerate a wide range of water temperatures (Favero et al. 2021). However, each species has an optimal temperature range for survival (generally between 22 ºC and 28 °C for Neotropical species), outside of which physiological stress and increased mortality may occur (Leira et al. 2017; Islam et al. 2022a, 2022b). Vicentini et al. (2024) reported that male *Rhamdia quelen* (Quoy and Gaimard, 1824) tolerates temperatures ranging from 15 ºC to 34 °C; however, individuals exposed to 30 °C showed a 15% increase in leukocyte infiltration and impairments in hepatic energy metabolism compared to those maintained at 25 °C. Similarly, numerous vacuole formations have been reported in *Ompok bimaculatus* (Bloch, 1794) exposed to 35 °C (Islam et al. 2022a, 2022b) and in *Clarias* spp., exposed to 32 °C and 37 °C (Khieokhajonkhet et al. 2022). In addition, *Clarias* spp. exhibited loss of cellular boundaries, irregularly shaped hepatocytes, and nuclear displacement, ultimately leading to hepatocyte degeneration (Khieokhajonkhet et al. 2022). For *A. lacustris*, temperature influences liver metabolism by increasing antioxidant defenses at higher temperatures, with temperatures between 26 °C and 28 °C ideal for cultivation of this species (Porto-Foresti et al. 2005; Siqueira-Silva et al. 2015; Ratko et al. 2022; Schleger 2022). To date, histological changes in the liver tissue of *A. lacustris* under elevated temperature conditions have not been described.

One way to evaluate the effects of elevated temperature on fish is through histological assessment of liver tissue, which reflects individuals' metabolic status in response to environmental changes (Yancheva et al. 2016). In addition, liver dysfunction may impair fish growth and reproduction, as the liver performs essential metabolic functions, including bile production, glycogen storage, plasma protein synthesis, detoxification of harmful substances, and the metabolism of lipids, carbohydrates, and proteins (Schleger 2022). This study aims to improve the understanding of key physiological responses by characterizing and evaluating potential changes in the liver histology of this species under elevated temperature conditions. Based on the projected increase in global temperature estimated by the IPCC (2014), we hypothesized that exposure to 32 °C would induce histological alterations in liver tissue and that the frequency of these alterations would increase with exposure duration. Specifically, we expected to observe changes associated with increased energy demand and metabolism, as well as enhanced antioxidant responses, including cytoplasmic vacuolization, leukocyte infiltration, loss of cellular boundaries, irregularly shaped hepatocytes, nuclear displacement, and hepatocyte degeneration. We characterized and histologically assessed liver tissue from fish maintained at optimal temperature (26 °C) and compared it with that of individuals exposed to elevated temperature (32 °C) for 30 and 210 days, as well as with that of individuals sampled at the beginning of the experiment. In addition, hepatosomatic and gonadosomatic indices were calculated and compared among treatments, considering the liver's role in reproduction.

## Materials and methods

### Experimental design and fish sampling

**Fig. 1:**
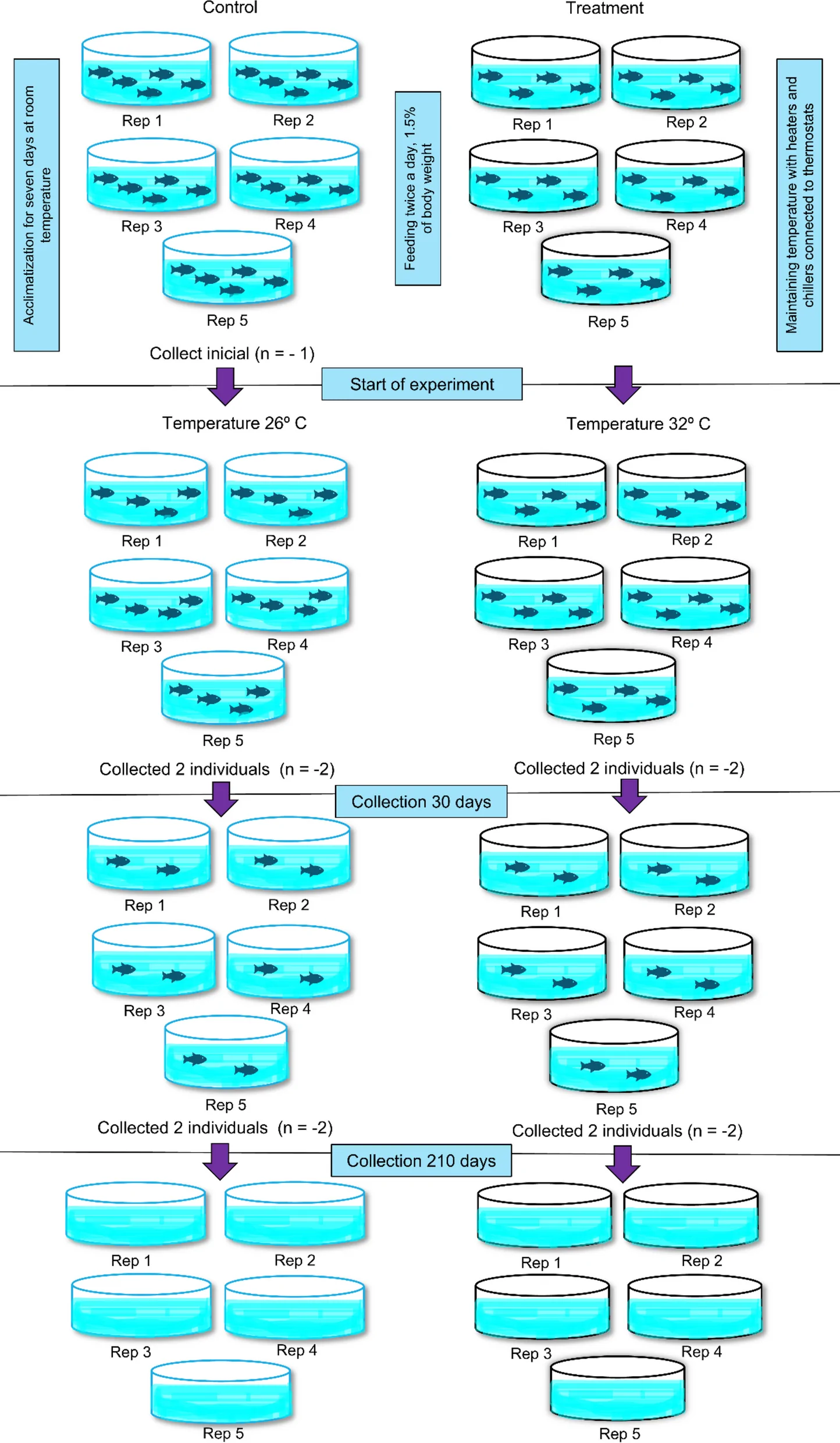
Experimental design. The experiment consisted of a control (26 °C) and a treatment (32 °C), where *Astyanax lacustris* was kept for 30 and 210 days. For each group, after the experiment began, 20 specimens were kept in four independent replicate tanks (replicates 1, 2, 3, and 4). At each sampling time (30 and 210 days), two fish were randomly sampled per replicate tank (*n* =  − 2), except for the initial collection, where only one individual (*n* =− 1) was sampled in the control group

Specimens of *A. lacustris* were obtained from the Centro de Aquicultura of São Paulo State University (UNESP), Jaboticabal, São Paulo, Brazil, and maintained in 1000 L tanks within a recirculating water system at the Laboratório de Ictiologia Neotropical, UNESP, Ilha Solteira, São Paulo, Brazil, for a period of seven months (September 2019 to April 2020). For this study, 45 male specimens were divided into two groups: a control group (*n* = 25) and a treatment group (*n* = 20). The specimens from these groups were distributed among 10 tanks, with five tanks assigned to each group, based on a biomass density of 62.5 g/m^3^ for each tank. In the control group (CT), each tank contained five specimens, whereas in the treatment group (TR), each tank contained four specimens (see Fig. 1 for the experimental design).

Initially, all groups were maintained at ambient temperature (mean: 26 °C) for seven days to allow acclimation to the recirculating system. After this period, five specimens were collected from the control group for initial biometry and liver morphological characterization. The CT group remained at ambient system temperature, while the TR group was subjected to a daily increase of 1 °C until reaching 32 °C (for six days), which was then maintained constant throughout the experiment. After 30 days of exposure to the respective temperature conditions, 20 specimens were randomly sampled from the 10 tanks, with two individuals collected per tank from each group (CT and TR). A final sampling of 10 specimens (two per tank from five tanks) was conducted after 210 days of exposure (see Fig. 1). Specimens were euthanized using a 0.01 g of benzocaine hydrochloride solution prepared in 1 mL of absolute ethanol and 1 L of water. After collection, the fish were immersed in this solution and remained there until they showed no signs of swimming or gill movement. Body mass was measured using an analytical balance (Marte®, model AY220). The liver and testis were then excised, weighed, and the liver was fixed in a solution containing 2.5% glutaraldehyde, 4% paraformaldehyde, and Sorensen’s phosphate buffer (pH 7.2) for 24 h.

Temperature variation in the CT group was monitored with a data logger. In the TR group, temperature was controlled using heaters and chillers connected to a thermostat. Fish were fed twice daily (1.5% of body weight) with commercial feed (Guabi-Pirámirin; 1.7 mm pellet size; 36% crude protein). To prevent the buildup of waste, the tanks were flushed once a week, and one-third of the water was replaced with water at the same temperature as the tank water. Water quality parameters, including pH and ammonia, were monitored and maintained within appropriate ranges for tropical fish species (Table 1; Ayroza 2011).

**Table 1:** Means (± standard deviation) of water quality parameters for tropical species subjected to natural conditions (26ºC) and thermal stress (32ºC). *According to Ayroza 2011

Measured parameters	26 ºC	32 ºC	Standards for tropical species*
Temperature (°C)	26.0 ± 0.1	32.0 ± 1.4	26 a 30 ºC
pH	7.4 ± 0.15	7.6 ± 0.06	6.0—9.0
Ammonia (NH_3_)	0.004 ± 0.004	0.003 ± 0.004	0.2 mg/L

All procedures for the maintenance, daily care, handling, and euthanasia of the animals were approved by the Ethics Committee on Animal Experimentation of the São Paulo State University//School of Engineering (10/2020—CEUA/FEIS).

### Liver histology

Fixed liver samples were dehydrated through a graded ethanol series, infiltrated, and embedded in glycol methacrylate resin (Historesin Leica®), which preserves tissue architecture without distortion. The embedded samples were mounted on support blocks and sectioned at 3 µm thickness using a microtome (Leica® RM 2244) equipped with a glass knife. Sections were mounted onto glass slides, with four sections per slide, and stained with hematoxylin and eosin (H&E) for general histological evaluation.

For histochemical analysis of glycogen, two slides per specimen were processed using the periodic acid–Schiff (PAS) method: 1% periodic acid for 15 min, rinsing in running water for 5 min, Schiff reagent for 40 min, followed by rinsing in running water for 10 min. Sections were then counterstained with Harris hematoxylin for 7 min and rinsed in running water.

For lipid detection, two slides per specimen were stained with Sudan Black B for 30 min, then gently rinsed in 70% ethanol and washed in distilled water. Sections were then counterstained with Rose Bengal for 1 min and rinsed again in distilled water.

Analysis and photodocumentation were carried out using a Zeiss Axio Scope A-1® optical microscope coupled with an Axiocam Mrc5® digital camera and an Olympus® optical microscope coupled with a Motic 2500® digital camera.

### Data analysis

For data analysis, the evaluated specimens were grouped by temperature (26 °C and 32 °C) and exposure period: initial (In), 30-day, and 210-day.

Histological alterations were quantified in five random microscopic fields/sections for four sections/samples. The incidence and distribution of liver alterations were evaluated based on the following criteria: absent (absence or lesions in up to 10% of the total tissue analyzed); rarely present (lesions in 11%–25% of the analyzed tissue); present (lesions in 26%–50% of the analyzed tissue); frequent (lesions in 51%–75% of the analyzed tissue); and very frequent (lesions in 76%–100% of the analyzed tissue) (Paulino et al. 2014). The histological alterations observed were classified into three progressive stages (Cerqueira and Fernandes, 2022 adapted from Poleksic and Mitrovic-Tutundzic 1994) according to degree of severity based on the possibility of the organ's normality and its recovery after environmental improvement: I, reversible alterations in which the normal structure of the organ can be recovered; II, alterations that are more serious and affect the functioning of the organ but may be repairable after environmental improvement; and III, alterations in which the restoration of the organ structure is not possible even after environmental improvement.

To compare the hepatosomatic index (HSI) between the groups and check whether there was a difference due to temperature and exposure period, the HSI (Vazzoler 1996) was calculated:

$$\mathrm{HSI}=\left(\text{liver mass/body mass}\right)\times100.$$

To relate the gonadosomatic index (GSI) to histological changes, the GSI (Vazzoler 1996) was calculated:

$$\mathrm{GSI}=\left(\text{gonad mass/body mass}\right)\times100.$$

The HSI and GSI values were analyzed using analysis of variance (ANOVA), followed by Tukey’s post hoc test, implemented with the “aov” and “TukeyHSD” functions in the *stats* package (R Core Team 2022). To do this, the data were log-transformed and submitted to the Bartlett homogeneity test and the Shapiro–Wilk normality test using the bartlett.test and shapiro.test functions from the ‘stats’ package (R Core Team 2022).

To assess the relationship between histological alterations and indices (HSI and GSI) in relation to exposure period and temperature, while accounting for individual body mass, a principal component analysis (PCA) was performed. We used the "prcomp" function from the stats package (R Core Team 2022). Through this analysis, it was possible to identify which variables best explained data variability and to assess correlations among variables based on the angles between vectors (smaller angle, greater correlation; Gotelli and Ellison 2016). Eigenvalues greater than one are considered for the extraction of the components (Kaiser criterion; Kaiser 1960). HSI, GSI, and histological alteration percentages were log-transformed for this analysis.

Statistical analyses were performed using R software (R Core Team 2022). The graph was prepared using the “ggplot2” package (Wickham 2016). The level of statistical significance was set at *p* = 0.05.

## Results

### Hepatic tissue characterization

**Fig. 2:**
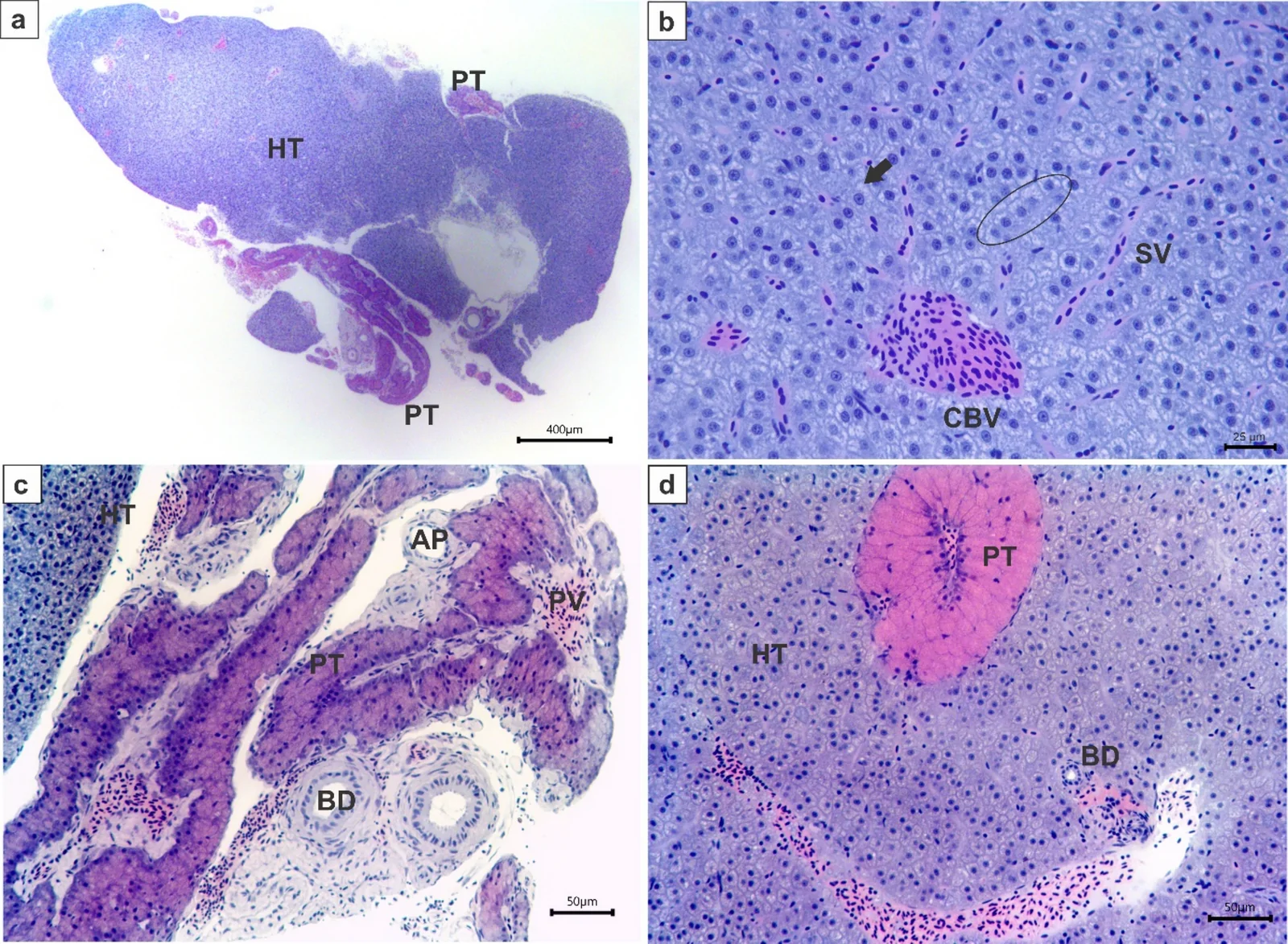
Characterization of the hepatic tissue (HT) of *Astyanax lacustris* in a water recirculation system at ambient temperature (26ºC). **a**. Longitudinal section of the liver demonstrating pancreatic tissue (PT) diffused in the hepatic tissue (HT). **b**. Hepatocyte cord (black circle) and hepatocyte (black arrow) are surrounded by blood capillaries (SV) with flow towards a central blood vessel (CBV). **c**. Portal space with bile duct (BD), portal artery (AP), and portal vein (PV) associated with pancreatic tissue (PT). **d**. Isolated bile duct (BD). Staining: Harris hematoxylin and eosin. Scale bars: **A**, 400 μm; **B**, 25 μm; **C**, 50 μm; **D**, 50 μm

The liver tissue of *A. lacustris* is composed of hepatocytes, sinusoidal capillaries, intrahepatic pancreas (hepatopancreas), portal triads (portal spaces), bile ducts, and blood cells (Fig. 2A–D). Hepatocytes are arranged in cords and exhibit a central nucleus, prominent nucleoli, and homogeneous cytoplasm (Fig. 2B). These cells are surrounded by irregularly dilated sinusoidal capillaries radiating from a central vein (Fig. 2B).

In association with pancreatic tissue, portal spaces composed of the portal vein, hepatic artery, and bile duct were observed (Fig. 2C). Bile ducts were identified by their simple cuboidal epithelium and occurred in two forms: associated with portal spaces (Fig. 2C) and as isolated ducts (Fig. 2D).

### Hepatic alteration

**Fig. 3:**
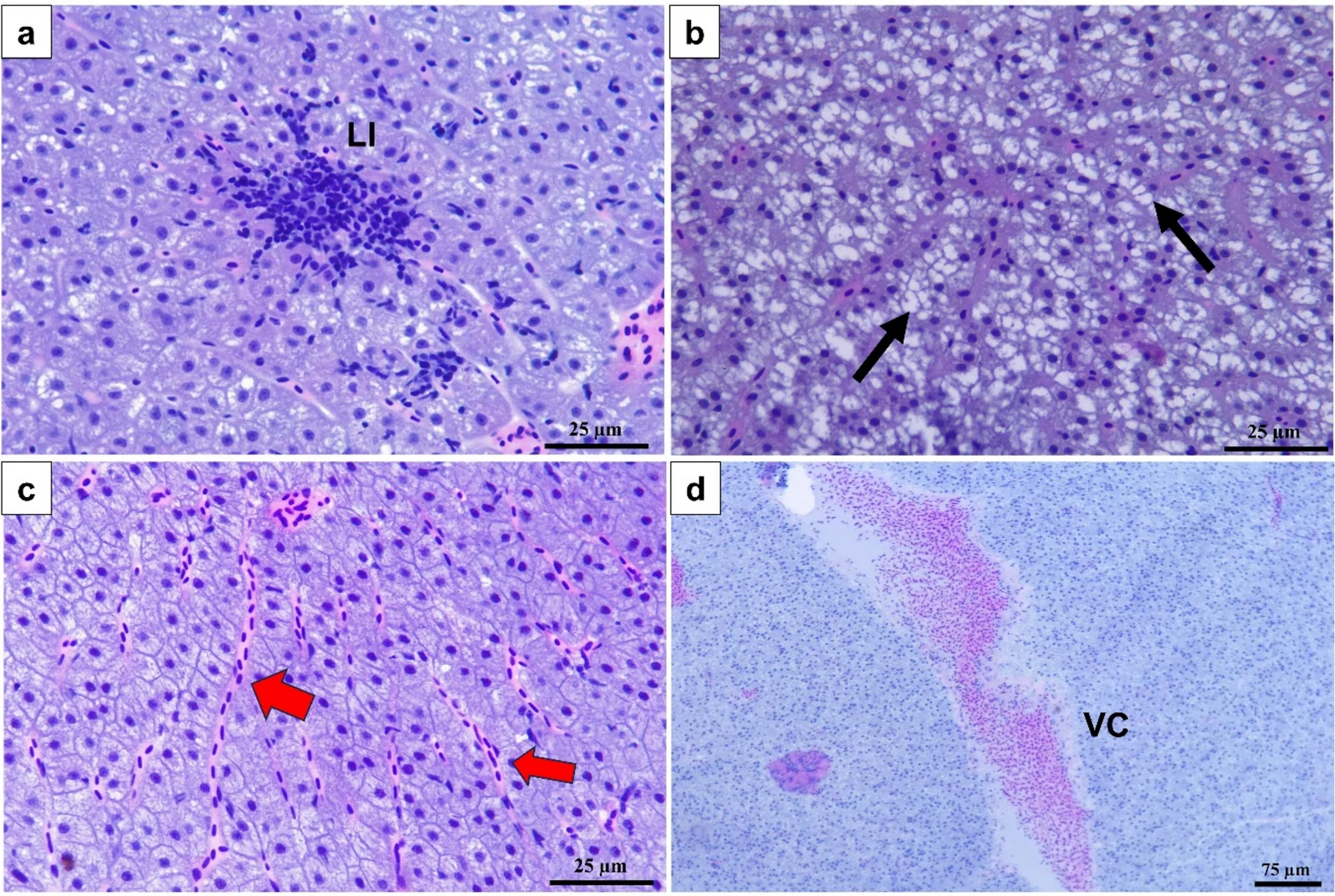
Histological alterations observed in the hepatic tissue of *Astyanax lacustris* in a water recirculation system. **a**. Leukocyte infiltration (LI); **b**. Cytoplasmic vacuolization (black arrow); **c**. Hyperemia (red arrow); **d**. Vascular congestion (VC). Staining: Harris hematoxylin and eosin. Scale bars: **A**, 25 μm; **B**, 25 μm; **C**, 25 μm; **D**, 75 μm

The histological alterations observed at stage I included leukocyte infiltration (LI; Fig. 3A), cytoplasmic vacuolization (black arrow; Fig. 3B), and hyperemia (red arrow; Fig. 3C), whereas stage II alterations included vascular congestion (VC; Fig. 3D). Overall, these alterations occurred at low frequencies and were classified as absent or rarely present (Table 2). No severe (stage III) alterations were observed.

**Table 2:** Average frequency of histological alterations observed in the hepatic tissue of *Astyanax lacustris* in each temperature and exposure time (AT- ambient temperature; In – initial collection). *indicates absence of data because there was no sampling for this group during this exposure period. LI—Leukocyte infiltration; CV—Cytoplasmic vacuolization; HY – Hyperemia; VC—Vascular congestion

Histological alterations	LI			CV			HY			VC			Weight (g)		
Stage	I			I			I			II					
Exposure time	AT	26º	32º	AT	26º	32º	AT	26º	32º	AT	26º	32º	AT	26º	32º
In	6.2	*	*	5	*	*	1	*	*	7.2	*	*	12.5	*	*
30	*	7.5	6.7	*	5	0	*	4.5	1.1	*	12.5	14.4	*	10.3	11.2
210	*	10	14	*	3.1	11.6	*	0.6	9.6	*	18.1	11.4	*	14.1	16.5

**Fig. 4:**
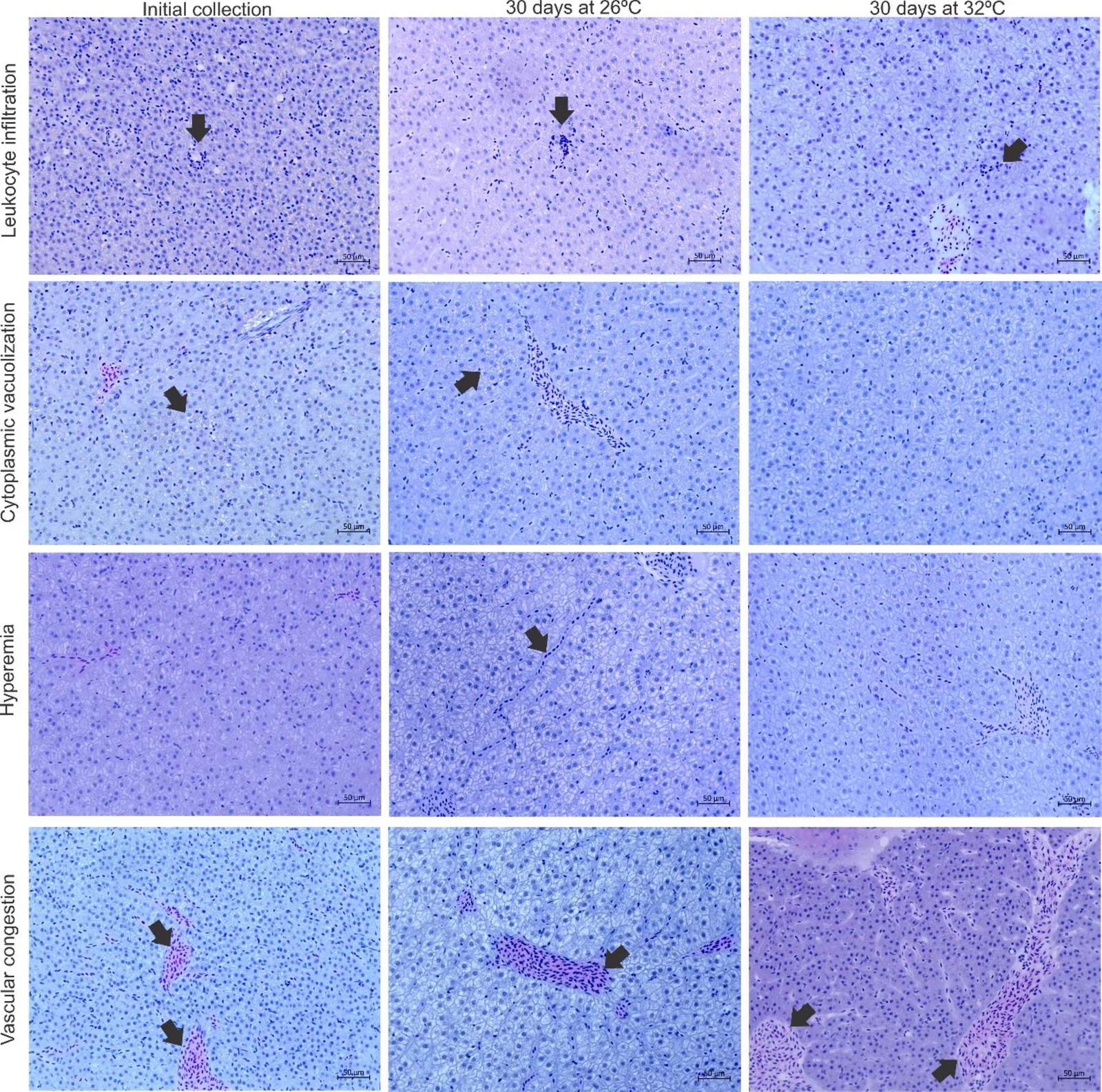
Comparisons of the histological alterations (indicated by a black arrow) observed in the liver tissue of *Astyanax lacustris* between the groups (IC: initial collection, CT30: 30-day control, TR30: 30-day treatment). The absence of an arrow indicates very low percentages (< 1.1) or no change in the group. Staining: Harris hematoxylin and eosin. Scale bars: All Figs. 50 μm

**Fig. 5:**
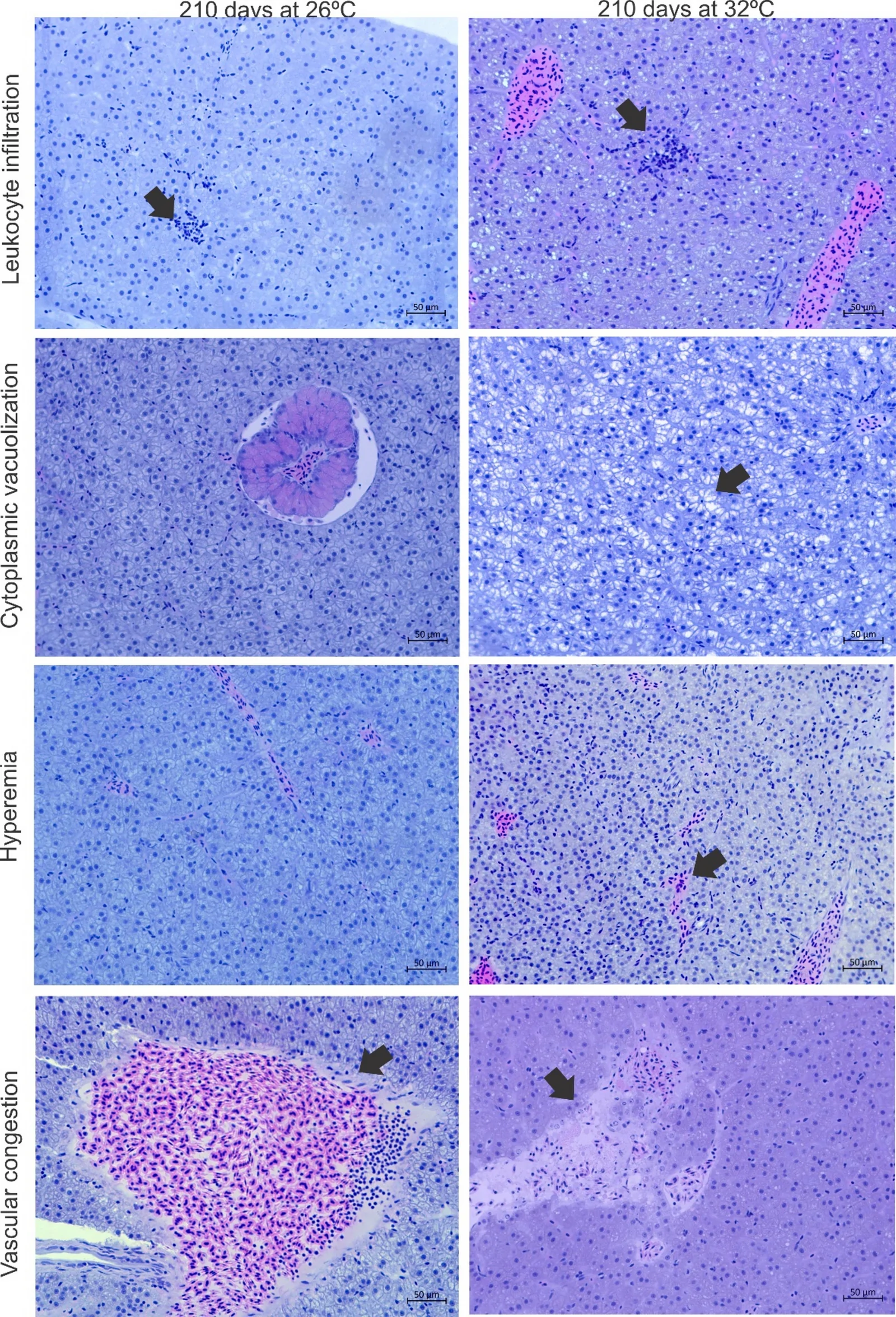
Comparisons of the histological alterations (indicated by a black arrow) observed in the liver tissue of *Astyanax lacustris* between the groups (CT210: 210-day control, and TR210: 210-day treatment). The absence of an arrow indicates very low percentages (< 1.1) or no change in the group. Staining: Harris hematoxylin and eosin. Scale bars: All Figs. 50 μm

**Fig. 6:**
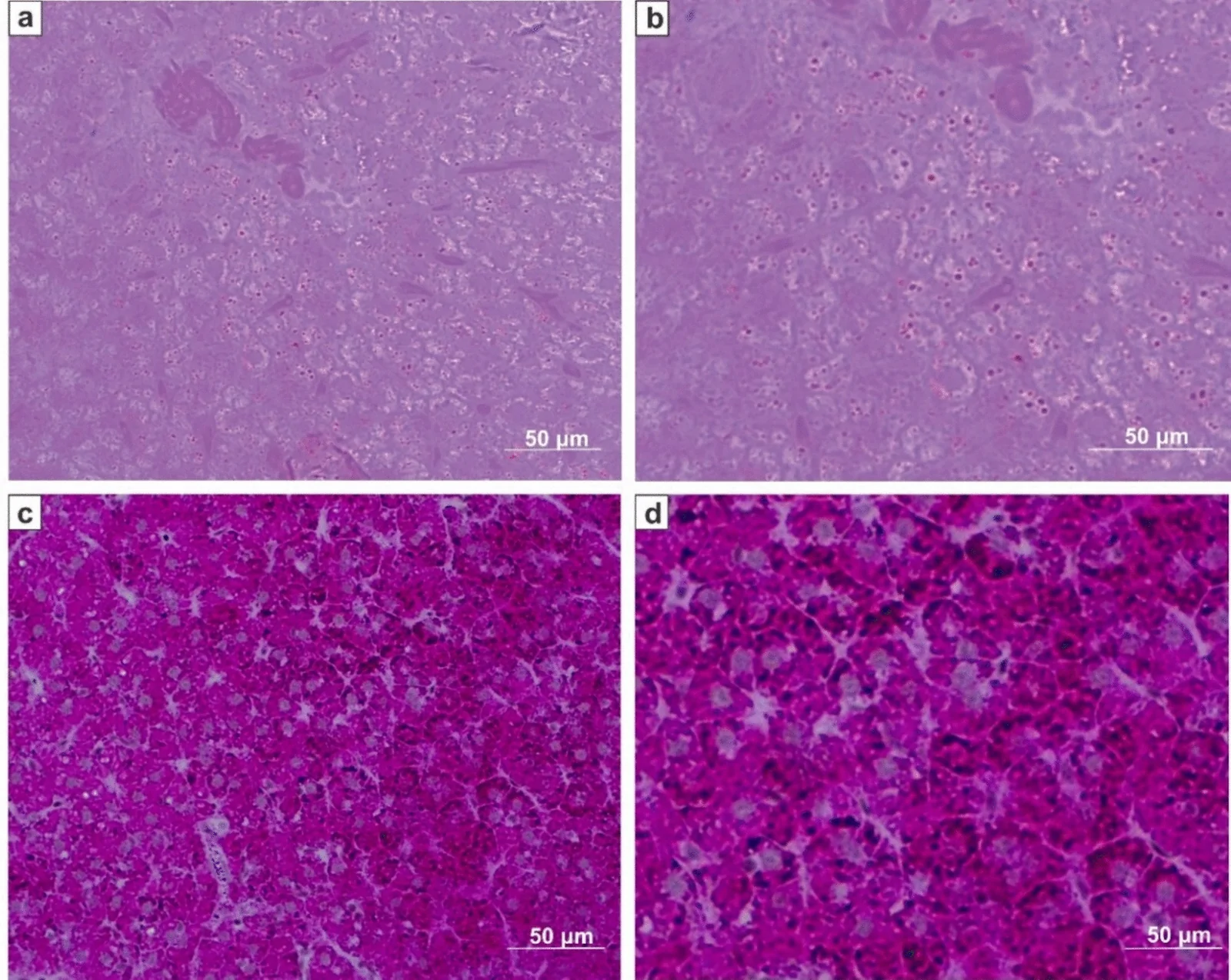
Histological liver sections of *Astyanax lacustris* submitted to the Sudan Black technique (**A** and **B**) and Periodic Acid Schiff (**C** and **D**) demonstrating the presence of lipids and glycogen in the vacuoles of hepatocytes. All Figs. 50 μm

Considering experimental groups, although they are classified as absent (absence or lesions in up to 10% of the total tissue analyzed) or rarely present (lesions in 11%–25%), the frequency of leukocyte infiltration, hyperemia, and cytoplasmic vacuolization increased with both temperature and exposure time (Table 2). Leukocyte infiltration was classified as absent in all groups except for the group exposed to 32 ºC for 210 days. Overall, leukocyte infiltration tended to increase with exposure time, regardless of temperature, with higher values observed in the 210-day groups (Table 2; Figs. 4 and 5). Cytoplasmic vacuolization was absent in all groups except in the group exposed to 32 ºC for 210 days, where it was classified as rarely present (Table 2; Figs. 4 and 5). These vacuoles were stained by Sudan Black and PAS, indicating both lipid (Fig. 6A, B) and glycogen content (Fig. 6C and D). Hyperemia was absent in most groups but occurred more frequently in the group exposed to 32 ºC for 210 days, increasing with exposure time (Table 2; Figs. 4 and 5). Vascular congestion was classified as absent at the beginning of the experiment and rarely present in other groups, with slightly higher occurrence in the group exposed for 210 days at 26ºC (Table 2; Figs. 4 and 5). These results suggest that vascular congestion was not directly associated with increased temperature.

The hepatosomatic index showed a slight increase at the beginning of the experiment, followed by a reduction over the exposure period, with the highest value observed in the group exposed for 30 days at 26 °C (Table 3). However, significant differences were detected only for the exposure period (DF = 1, F = 4.55, *p* = 0.04), driven by differences between the initial sampling and the other periods (In vs. 30 days: *p* = 0.01; In vs. 210 days: *p* = 0.03). No significant differences were observed between groups (DF = 2, F = 2.02, *p* = 0.15) or in the interaction between group and exposure period (DF = 1, F = 0.19, *p* = 0.66).

**Table 3:** Hepatossomatic index and Gonadosomatic index observed in the hepatic tissue of *Astyanax lacustris* at each temperature and exposure time (AT—ambient temperature; In – initial collection). *indicates absence of data because there was no sampling for this group during this exposure period

	Hepatossomatic index			Gonadosomatic index		
Exposure time	AT	26º	32º	AT	26º	32º
In	0.6	*	*	2.5	*	*
30	*	0.9	0.8	*	3.4	3.8
210	*	0.8	0.7	*	2.88	1.9

The gonadosomatic index showed the lowest values in the group exposed to 32 °C for 210 days and the highest values in the group exposed for 30 days (Table 3). However, significant differences were observed only for the exposure period (DF = 2, F = 6.41, *p* = 0.004), driven by differences between 30 days and the other periods (In vs. 30 days: *p* = 0.04; 30 vs. 210 days: *p* = 0.002). No significant differences were detected between groups (DF = 1, F = 0.44, *p* = 0.50) or in the interaction between group and exposure period (DF = 1, F = 3.08, *p* = 0.08).

**Fig. 7:**
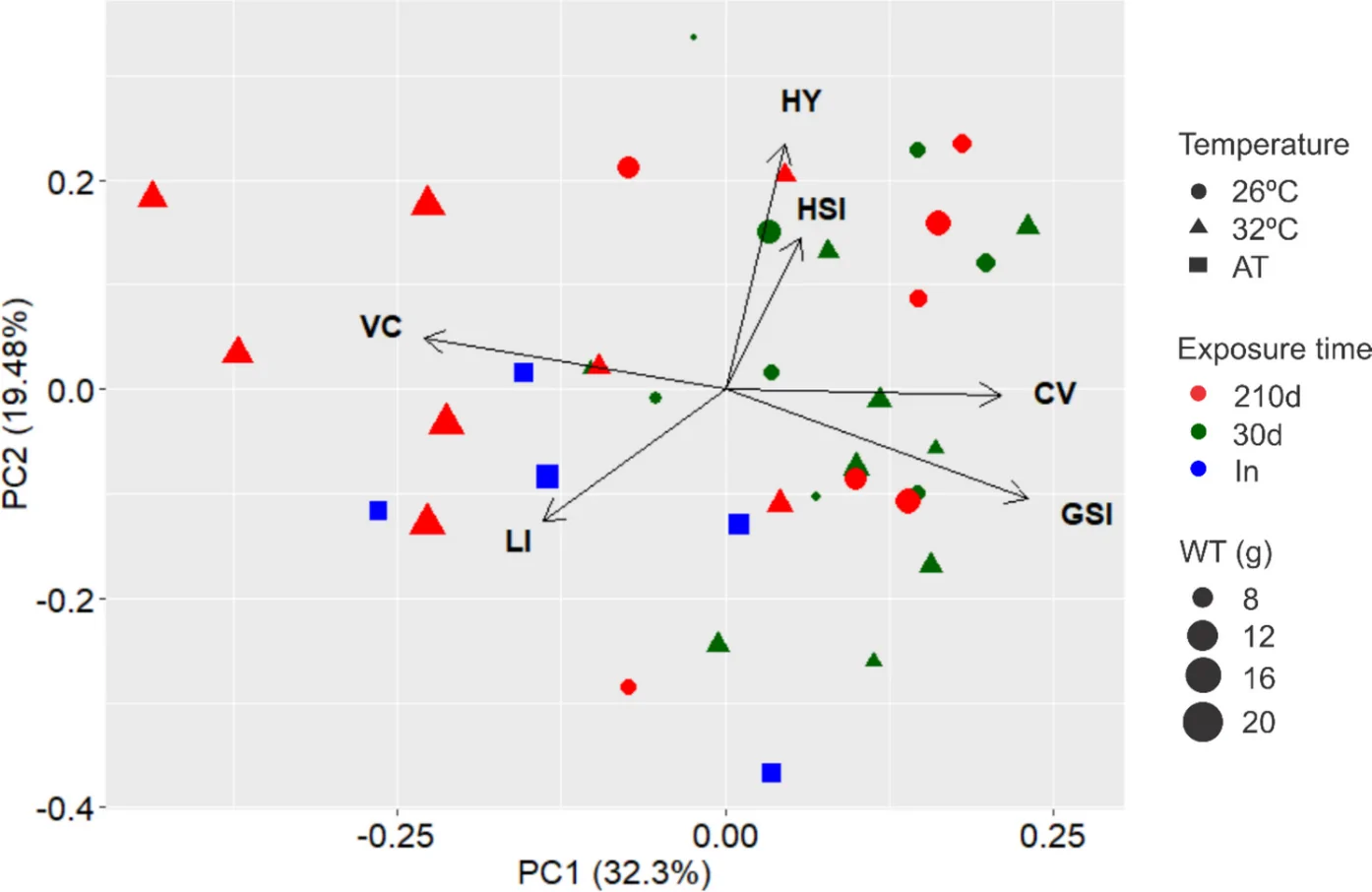
Biplot of the principal component analysis (PCA), showing the relationships between histological changes, the hepatosomatic (HSI) and gonadosomatic indices (GSI) for *Astyanax lacustris,* considering the weight (WT) of individuals, the water temperature, and the exposure period (ExP). LI: Leukocyte infiltration; HY: Hyperemia; CV: Cytoplasmic vacuolization; VC: Vascular congestion; In = initial collection; AT = ambient temperature

Principal component analysis (PCA) revealed that the first two axes explained 51.8% of the total data variability, with PC1 accounting for 32.3% and PC2 for 19.5% (Fig. 7; Table 4).

**Table 4:** Results of Principal Component Analysis (PCA) applied to histological changes and indices (HIS and GSI) between groups to verify the relationship with exposure period and temperature, considering individual weight

	PC1	PC2
Histological alterations		
Cytoplasmic vacuolization	0.76	−0.34
Leukocyte infiltration	−0.46	−0.42
Hyperemia	0.15	0.78
Vascular congestion	−0.76	0.16
Index		
Gonadossomatic index	0.76	−0.34
Hepatossomatic index	0.19	0.48
Variance (%)	32.3	19.48
Eigenvalue	1.93	1.16

PC1 separated individuals mainly along a gradient associated with CV and GSI on the positive side, and VC and LI on the negative side. Individuals from the 30-day groups (especially at 32 °C) were predominantly distributed on the positive side of PC1, indicating association with higher CV and GSI values. In contrast, individuals from the 210-day groups were more widely distributed, with several positioned on the negative side of PC1, associated with higher VC and LI values. Additionally, individuals with greater body mass were more frequently associated with this negative portion of the axis.

PC2 was mainly influenced by HY and HSI, which were positively correlated with this axis. Individuals with higher HSI and HY values were located in the upper portion of the plot, regardless of temperature. Conversely, individuals associated with higher LI and GSI values tended to occupy the lower portion of PC2.

Overall, the PCA indicated that blood flow alterations, such as HY and VC, were associated with indices (HSI and GSI) related to the reproductive cycle. In contrast, CV was clearly associated with increased temperature and longer exposure time.

## Discussion

The histological characterization of the liver revealed that hepatocytes, sinusoidal capillaries, intrahepatic pancreas, portal spaces, and bile ducts are fundamental for organ function (Junqueira and Carneiro 2013; Mokhtar 2017) and are consistent with descriptions in other teleosts (Bombonato et al. 2007; Costa et al. 2015). These structures support key hepatic functions, including nutrient assimilation, bile production, detoxification, and metabolic homeostasis (Genten et al. 2009). Hepatocytes play a central role in liver regeneration and in metabolic processes such as biotransformation, lipid metabolism, nutrient storage, and bile salt production (Schinoni 2006; Buzato et al. 2016; Chen et al. 2020). The intrahepatic pancreas produces digestive enzymes and is diffusely distributed, often associated with portal spaces (Bombonato et al. 2007; Genten et al. 2009). Portal spaces form portal triads composed of a vein, an artery, and a bile duct (Mokhtar 2017). In *A. lacustris*, bile ducts were observed both as isolated structures and associated with portal spaces, as described for other teleosts (Akiyoshi and Inoue 2004).

Our results showed that fish exposed to 32 °C exhibited histological alterations in liver tissue, confirming our hypothesis. Based on previous studies in other species (Khieokhajonkhet et al. 2022; Islam et al. 2022a, 2022b), we expected to observe alterations such as cytoplasmic vacuolization, leukocyte infiltration, loss of cellular boundaries, irregularly shaped hepatocytes, nuclear displacement, and hepatocyte degeneration. However, only leukocyte infiltration, cytoplasmic vacuolization, vascular congestion, and hyperemia were observed, all occurring at low frequencies and without evidence of functional impairment of liver tissue. Additionally, the higher GSI values observed in *A. lacustris* (indicator of the reproductive cycle as demonstrated by Ugrin et al. 2023; Ludwig et al. 2024 and Chen et al. 2026) were associated with cytoplasmic vacuolization and hyperemia, suggesting that these alterations may be linked to changes in blood flow during the reproductive period (Rizzo and Bazzoli 2020).

Histological alterations were identified by comparing liver tissue between the control and treatment groups. Although these alterations were classified as stage I, occurred at low frequency (absent or rarely present), and did not impair normal liver function (Cerqueira and Fernandes 2022), trends toward increased temperature and exposure time were observed. Leukocyte infiltration, cytoplasmic vacuolization, and hyperemia increased with both temperature and exposure duration, indicating responses to thermal stress, as also reported by Islam et al. (2022a, 2022b), Khieokhajonkhet et al. (2022), and Vicentini et al. (2024).

Leukocyte infiltration was more frequent in the 210-day groups, particularly in 32ºC, indicating an association with exposure time and increased temperature. This alteration reflects inflammatory responses and hypersensitivity in the affected tissue, likely related to thermal stress (Bernet et al. 1999; Nimet 2016). Similar findings were reported by Vicentini et al. (2024) in *R. quelen* exposed to 30 °C.

Cytoplasmic vacuolization, which was also more frequent in the group exposed to 32 ºC for 210 days, is a common feature in captive fish and may be associated with multiple factors (Roberts 2012). In many cases, moderate to high vacuolization reflects lipid or carbohydrate accumulation due to excess energy intake under confinement (Wolf and Wheeler 2018). However, as feeding was controlled in this study, the low frequency of vacuolization suggests that it was not driven by dietary excess. Instead, our results indicate that metabolic disturbances associated with elevated temperature may explain this pattern. Temperatures above the optimal range can increase hepatic glucose levels (Schleger 2022) and disrupt lipid metabolism (Lee et al. 2012; Nimet 2016), leading to the formation of cytoplasmic vacuoles due to lipid and glycogen accumulation. Similar temperature-related alterations have been reported in *Clarias* spp. (Khieokhajonkhet et al. 2022), *Megalobrama amblycephala* (Liu et al. 2016) and *Paralichthys olivaceus* (Liu et al. 2015).

Hyperemia was more frequent in the group exposed to 32 ºC for 210 days and is characterized by increased blood flow within a given tissue (Bernet et al. 1999; Paulino et al. 2014). This alteration may represent an adaptive response to environmental stressors, such as elevated temperature (Silva 2004). However, the association between HY and HSI and GSI in the PCA suggests that this response may also be linked to the reproductive period, as observed for VC. In contrast, VC showed no clear relationship with temperature or exposure time, as it was already present, albeit at low frequency, after 30 days. This pattern reinforces its association with reproductive processes rather than thermal stress. Vascular congestion occurs when blood flow is impaired or obstructed, leading to vessel dilation, reduced circulation, and potential tissue damage due to coagulation and epithelial disruption (Verlag 1982; Thomson 1983). In the context of the reproductive period, an increase in blood flow to the liver of male fish is expected as a fundamental physiological response to support the high energy demands of spermatogenesis and the production of seminal plasma components (Baldisserotto et al. 2020). The reproductive period of *A. lacustris* runs from September to the end of March, which coincides with the experimental period of this study (Porto-Foresti et al. 2001).

The hepatosomatic index (HSI) represents the proportion of liver mass relative to body weight and is commonly used as an indicator of hepatic energy reserves (glycogen) and overall physiological condition (Cyrino et al. 2000; Baldisserotto and Gomes 2013). This index is often associated with the reproductive period (Querol et al. 2002). However, the differences in HSI observed between initial collection and the group 30 days at 26ºC, and between initial collection and the group 30 days at 32ºC, suggest that additional factors, such as handling stress and the initial period of confinement, may have influenced these values. As a highly sensitive organ, the liver responds rapidly to environmental changes (Yancheva et al. 2016; Yeşilbudak 2024), indicating that HSI may reflect metabolic adjustments associated with stress induced by external factors.

In conclusion, as predicted by our hypothesis, the liver of *A. lacustris* exhibited increased frequencies of histological alterations under elevated temperature (32 °C) and prolonged exposure. Cytoplasmic vacuolization, leukocyte infiltration, and hyperemia increased with exposure time, indicating progressive physiological responses to thermal stress. However, the low frequency and mild severity of these alterations suggest that exposure to 32 °C for up to 210 days does not result in functional impairment of liver tissue.

From an applied perspective, these findings indicate that *A. lacustris* can tolerate elevated temperatures under controlled recirculating aquaculture systems without overloading liver function. Nevertheless, the observed trends highlight that prolonged exposure may lead to more pronounced alterations, particularly under conditions that combine thermal stress with other environmental or physiological factors. Thus, future warming scenarios in natural ecosystems may impose greater risks, reinforcing the importance of long-term assessments when evaluating the impacts of climate change on fish health and aquaculture sustainability.

## Data Availability

Data will be made available on request.
